# Prevalence and Incidence of Smear-Positive Pulmonary Tuberculosis in the Hetosa District of Arsi Zone, Oromia Regional State of Central Ethiopia

**DOI:** 10.1186/s12879-017-2321-0

**Published:** 2017-03-16

**Authors:** ShalloDaba Hamusse, Meaza Demissie, Dejene Teshome, Mohammed Suaudi Hassen, Bernt Lindtjørn

**Affiliations:** 1 Oromia Regional Health Bureau, Addis Ababa, Ethiopia; 2 Addis Continental Institute of Public Health, Addis Ababa, Ethiopia; 3 Adama Regional Research Centre Laboratory, University of Bergen, Bergen, Norway; 4 Centre for International Health, University of Bergen, Bergen, Norway

**Keywords:** Pulmonary smear-positive TB, Prevalence, Incidence, Arsi Zone, Ethiopia

## Abstract

**Background:**

The real burden of smear-positive (PTB+) and bacteriologically confirmed tuberculosis (BCTB) in Ethiopia is not known. Thus, the aim of this community-based study was to measure the prevalence and incidence of tuberculosis in the Hetosa District of Oromia Region, Ethiopia.

**Methods:**

First, a population-based cross-sectional survey was conducted on a total of 33,073 individuals aged ≥ 15 years to determine the prevalence of PTB+ and BCTB cases. Then, in order to determine the incidence, a prospective follow-up was carried out on 32,800 individuals found to be either free from symptoms suggestive of TB (SSTB) during the baseline survey or had symptoms suggestive of TB but yielded negative bacteriological examination results. We identified 1,041 presumptive TB cases at the baseline survey, and 1,468 in the follow-up study. Each participants with cough of more than two weeks were provided spot and morning sputum samples for acid-fast bacilli sputum microscopy and culture.

**Results:**

At the baseline survey, 43 BCTB cases were identified. Thirty six of these were both smear- and culture-positive while seven were only culture-positive. In the follow-up study, however, 76 BCTB cases were diagnosed and 70 of these were found to be both smear- and culture-positive while six were culture-positive only. The adjusted prevalence of PTB+ and BCTB in the study area was 109 and 132/100,000 persons, respectively. Moreover, the incidences of PTB+ and BCTB were 214 and 232/100,000 persons per year (py), respectively. The ratio of the passive to active case finding was 1:0.96 (45/43). For every TB case identified through the existing passive case diagnosis, there was an almost equal number (0.96) of undiagnosed infectious TB cases in the community. A family history of TB contact was independently associated with a high risk of TB (TB prevalence, AOR, 13; 95% CI: 6.55–15.33) and (TB incidence, aIRR 4.11, 95% CI: 2.18–7.77).

**Conclusions and recommendations:**

The prevalence and incidence of smear-positive and bacteriologically confirmed TB cases were high in the study area. For every case of smear-positive TB receiving treatment, there was an almost equal (0.96) number of undetected infectious bacteriologically confirmed TB case in the community. The high proportion of undetected infectious TB cases in the community could possibly be due to the sub-optimal performance of Directly Observed Treatment Short-course (DOTS) in detecting 70% of infectious TB cases, as well as attaining a cure rate of 85% in the study area. Family history of TB contact has substantaially increased the risk of developing the disease, and there is a need to improve ways of identifying TB cases and intensify mechanisms of tracing contacts among household members of PTB+ cases.

## Background

Even though highly effective first-line short-course regimens that can cure about 90% of tuberculosis (TB) cases have been available for decades, the disease remains a major cause of morbidity and the second leading cause of death worldwide. Only in 2014,there were an estimated of 9.6 million TB incidents and 1.5 million deaths due to the disease worldwide [1–3].

In 1993, the World Health Organization (WHO) declared TB to be a global public health emergency and in 1994 formally launched the Directly Observed Treatment Short-course (DOTS) as a standard strategy to control the disease [4]. Since then significant progress has been made in reversing the incidence of TB and it was possible to reduce its prevalence by 41% worldwide [5]. However, in sub-Saharan Africa and other resource-constrained countries, the number of new TB cases reported is steadily increasing. Moreover, 80% of TB cases and 78% of global TB deaths occur in these countries, primarily due to the high prevalence of human immuno-deficiency virus (HIV), poor TB control efforts, social inequalities, drug resistance and inadequate access to TB care [4–6].

The incidence and prevalence of TB are among the valuable epidemiological indicators used to measure the impact of TB control efforts and assess the progress made towards the Millennium Development Goals (MDGs) [7–9]. A recent systematic review has shown that the current fixed value of the annual risk of TB infection derived using the Styblo rule in the estimation of TB incidence in the community is no longer valid in the era of the HIV epidemic due to the fact that the incidence of TB cases is fuelled by the powerful interaction between tuberculosis and HIV [9, 10]. Hence, the true TB incidence and prevalence in the community could only be obtained through population-based surveys and prospective follow-up studies that measure the impact of TB control efforts in a particular country [8]. However, these types of data are lacking in developing countries including Ethiopia [11, 12].

Moreover, estimating the incidence of TB is a challenge due to the fact that enrolling many people in a prospective follow-up study is difficult [8]. As a result, two consecutive community-based prevalence surveys within a short time interval is an alternative option [8]. However, deriving TB incidence from prevalence surveys requires a good estimate of disease duration, which is difficult to obtain from such surveys. In general, the true TB incidence can be measured by either conducting a prospective follow-up study or by carrying out two consecutive prevalence surveys within a short time interval, and then estimating the number of new TB cases that occurs between the surveys [7–9].

According to the global TB report of 2015, the TB prevalence and incidence in Ethiopia were estimated at 190 (95% CI: 160–240) and 200 (95% CI: 160–240), respectively [1]. Moreover, the 2011 National TB Prevalence Survey and other reports from different parts of the country showed that the TB prevalence ranged between 30 and 213.4 per 100,000 population [12–18]. However, the real burden of smear-positive pulmonary TB and bacteriologically confirmed TB cases in Arsi Zone, in general, and Hetosa District, in particular, was not known. Thus, the aim of this study was to measure the prevalence of bacteriologically confirmed pulmonary TB at the baseline survey, and then to investigate the incidence of TB through a prospective follow-up study in the Hetosa District of Arsi Zone, Central Ethiopia.

## Methods

### Study setting

The Hetosa District is one of the 25 districts of Arsi Zone, Oromia Regional State of Central Ethiopia. The district is typical of the zone in terms of population density, socio–cultural and economic state, and demographic conditions. Therefore, it is expected that the results could be generalized to the whole zone. Based on the 2007 Census Projection, the district has an estimated population of 178,229 living in one urban and 23 rural *kebele*s (the smallest administrative unit in the government structure) where an average of 2.7 adults were living in each household [19]. Since 2010, each *kebele* in the district has been further divided into six sub-*kebele*s in the rural areas and 10 sub-*kebele*s in the urban centres. The sub-*kebele*s are known as *garee*s. The *garee*s of the district total to148 (138 from rural and 10 from urban *kebele*s) [20]. This study was carried out in 49 randomly selected *garee*s (clusters).

### Study design and population

A population-based cross-sectional survey using multi-stage cluster sampling method was used to estimate the prevalence of smear-positive TB (PTB+) and bacteriologically confirmed TB (BCTB) cases at baseline. Next, a prospective follow-up study design was employed to estimate the incidence of the disease. In the estimation of PTB+ and BCTB incidence, individuals who were free of a persistent cough for more than two weeks, fever, loss of appetite, weight loss, blood-stained sputum and chest pain or difficulty in breathing [symptoms suggestive of TB (SSPTB)] at baseline, and those who had SSPTB at baseline but later showed negative result in a bacteriological test, were adopted as a cohort for the prospective follow-up study. The study was carried out from July 2013 to June 2014.

The source population for the study were adult individuals aged ≥15 years and permanently living in the district. Eligibility criteria were age ≥15 years, willingness to provide written consent to participate in the study, and a permanent residence for at least 15 days in the selected house prior to the start of the study. Additionally, participants had to be individuals with SSPTB.

### Sample size and sampling techniques

For economic and practical reasons, and because it is typical of the whole of Arsi Zone, the Hetosa District was purposefully selected from the 25 districts in the zone. All the 23 rural and one urban kebeles of the district were included in the study. The number of eligible population in the urban kebele was 5,903, and the population in the 23 rural kebeles was 27,170. Moreover, 18 of the rural kebeles have higher population density compared to the remaining five. Each of the former kebele has about double the population size of each of the latter. The number of clusters (garees) allocated to each urban and rural kebele was proportional to its population size. Consequently, a stratified multi-stage random sampling procedure was used to select two garees from each of the 18 rural kebeles high population density, and one garee from each of the remaining five rural kebeles. Moreover, based on the urban population of 5,903 and rural population of 27,170 that fulfilled the eligibility criteria, eight garees from the urban and 41 from the rural kebeles were included in the study [20]. An alphabetically arranged list of garees in each kebele along with their population size was obtained from the district authorities. Subsequently, garees were randomly selected from the list and included in the study. All individuals aged ≥15 years residing in the selected garees were included in the study.

The prevalence of PTB+ used in the sample size calculation was 382 per 100,000 based on the assumption that has been used in the 2011 national prevalence survey of the adult population aged ≥15 years [21], and an estimated 210/100,000 in 2012 for Ethiopia by the WHO [22]. Consequently, we calculated the sample size using a prevalence of 382 per 100,000, a relative precision of 0.25, an expected participation rate of 90% and a design effect of 2, a sample size of 33,448 people from 49 clusters. However, in the house-to-house enumeration held during the pre-survey of all the 49 selected *garee*s, 34,707 adults aged ≥ 15 years were identified. As the number was very similar to that of the calculated sample size, we enrolled all of them in the study.

### Data collection procedures

The aims of the study and the procedures for data collection were discussed with zonal, district and *kebele* leaders. The District TB Coordinator selected 24 nurses and 24 health extension workers (HEWs) for data collection, five laboratory technicians for sputum sample collection and 10 health officers for supervision. A health extension worker (HEW) is a female community health worker trained for one-year and deployed at a *kebele* with the responsibility of providing essential health services to ensure equitable access to health care, prevent major communicable diseases and promote health in the community [23]. Altogether, a total of 48 data collectors, five laboratory technicians and 10 supervisors were trained on TB screening techniques and on how to collect and transport sputum specimens. As on average about 2.7 adult were living in each household, each data collector was responsible to interview for about 11 to 12 study participants from four to five households with in a day.

The baseline survey to determine the prevalence of PTB+ and BCTB was conducted in May to June 2013. Subsequently, the prospective follow-up study to determine the incidence of PTB+ and BCTB was carried out between July 2013 and June 2014. Individuals who met the eligibility criteria, and were willing to provide written consent to participate in the study were included.

Study participants with SSPTB were identified as presumptive TB cases and were interviewed about their age, sex, history of contact with known TB patients and any current or previous TB treatment both at the baseline survey and prospective follow-up study. Participants with any SSPTB were requested to submit two sputum samples, one on the spot and the other in the morning of the following day. Upon receipt from the participants, the specimens were immediately put in sterile flacon tubes and placed in a cold box at 4°C and transported on the same day to the Adama Regional Research Centre Laboratory.

The following day each smear was fixed, air-dried and stained using the standard Ziehl-Neelsen (ZN) methods [24] and examined by experienced laboratory technicians for the presence of acid-fast bacilli (AFB). Positive results were quantified using the International Union against Tuberculosis and Lung Disease (IUATLD) standards [25]. A senior laboratory technologist blinded to the first test results re-examined all the smear-positive and 10% of the smear-negative slides. However, no discordant test results were observed between the two examinations. Moreover, sputum cultures using Lowenstein-Jensen (LJ) medium were commenced within a maximum of two days from the receipt of the sputum. In the event the diagnostic test did not commence on the 2nd day following specimen collection, the sputum sample was stored at −20°C in the same laboratory until tests were undertaken.

The data collectors also checked TB patient registration units in the study area to verify that those who reported they were on anti-TB treatments at the time of the study were actually on medication, and if there was any patient who was on anti-TB medication but did not report it during the survey period. However, no mismatch was identified.

Pulmonary smear-positive TB (PTB+) is defined as a patient found to be positive for AFB in both spot and morning sputum samples examined using direct microscopy or a patient found to be smear-positive in either spot or morning sputum examinations for AFB and culture-positive. Further, bacteriologically confirmed TB (BCTB) cases are individuals with smear- and/or culture-positive results. Types of TB were defined based on the 2011 WHO Tuberculosis Prevalence Survey Handbook [26] as follows:

#### New case not on treatment

A patient who has never received TB treatment for more than a month and who is not being treated currently with any anti-TB drugs.

#### New case on treatment

A patient who is currently being treated with anti-TB drugs, but has previously not received any anti-TB treatment for more than a month.

#### Previously treated case not on treatment

A patient who has previously received treatment for TB for more than a month, but who is currently not receiving any treatment with anti-TB drugs.

#### Previously treated case on treatment

A patient who has previously received treatment for TB for more than a month and who is currently being treated with anti-TB drugs.

The sputum smear-positive results were communicated through both written and telephone reports to TB focal persons at health centres in the study sites. The diagnosed TB cases started anti-TB treatment according to the national TB guidelines [24], with a culture performed on morning specimens using Lowenstein-Jensen (LJ) medium. The results were considered to be negative if no colonies were identified after eight weeks of incubation. Positive results from the LJ cultures were confirmed by testing for the presence of AFB through microscopic examination using the Ziehl-Neelsen method.

In the follow-up study, a total of 32,800 individuals who were free from SSPTB at baseline study and those with SSPTB but negative bacteriological results during the same survey were followed up for 12 months (July 2013 to June 2014) so as to estimate the incidence of PTB+ and BCTB cases. At intervals of six months, both at the end of the sixth and the 12th months from the baseline study, the same data collectors revisited all households that had been visited at the baseline and interviewed each person aged ≥15 years. The same data collection procedure, sputum sample collection, laboratory testing procedures and questionnaire were used in the prospective follow-up study. To ensure the data quality, the principal investigator and supervisors closely monitored the data collection process.

### Data entry and analysis

All data collected using the standardized and pre-tested questionnaire were coded and double-entered into Epi-info version 7 statistical software. The data were checked against the original questionnaires for missing variables, and errors were corrected by referring to the original questionnaires. Data analyses were performed based on the method recommended by the WHO Tuberculosis Prevalence Survey Handbook for the estimation of PTB+ and BCTB prevalence [26]. In the initial model of analysis, the crude PTB+ and BCTB prevalence was estimated without taking into account the sample cluster survey design effect. Nonetheless, in the final model, a complete analysis with an inverse probability weighting was carried out using robust standard errors to account for the sample cluster survey design effect, and the adjusted estimated prevalence of both PTB+ and BCTB were computed and reported [26]. Data analysis was carried out using STATA (v12.1, Stata Corporation, College Station, TX, USA).

Furthermore, in the analysis of TB incidence, a persons per year observation (pyo) was used as a denominator where person-time at risk of TB began in June 2013 when eligible individuals started participating in the study. Enrolment ended when participants were found to be AFB and/or culture-positive and were censored in June 2014. However, as the exact time of contribution of those who dropped out in the course of the study due to out-migration or death was not known, we excluded 185 participants (17 deaths, 47 refusal cases and 119 out-migrants) from the analysis to avoid bias due to ambiguity surrounding the time of their contribution. In order to avoid an under estimation of TB incidence, the principal investigator checked all health facilities in the study area to verify whether any TB cases were diagnosed and registered during the 12 months of the follow-up study, but none was found.

The prevalence and incidence of PTB+ and BCTB were taken as the dependent variables whereas age, sex, area of residence and family history of TB contact were the independent variables. The independent and dependent variables were further categorized into groups for analysis. A Poisson regression analysis was carried out in the analysis of TB incidence. The estimated incidence rate ratios (IRRs) and adjusted odds ratio (AOR) at 95% confidence intervals (CI) and *P*-values of less than 0.05 were used to assess the strength of association with PTB+ and BCTB cases as the outcome.

### Ethical considerations

The study protocol was reviewed and approved by both the Regional Committee for Medical and Health Research Ethics in Western Norway (REK Vest) and the Institutional Review Board Committee at the Oromia Health Bureau, Ethiopia. All participants were informed that taking part in the study was based fully on their willingness, and that they had the right to quit at any time from the study. Before any interview started, in the prevalence survey and in the subsequent follow-up study written consents were obtained from all participants aged ≥18 years and from parents/guardians if participants were <18 years of age. Data on individuals were analysed and anonymously reported. Immediate referrals were arranged for participants found to be smear or culture-positive, and all started anti-TB treatment at health centres close to them. The principal investigator also confirmed that all patients started treatment.

## Results

### Survey population

**Fig. 1:**
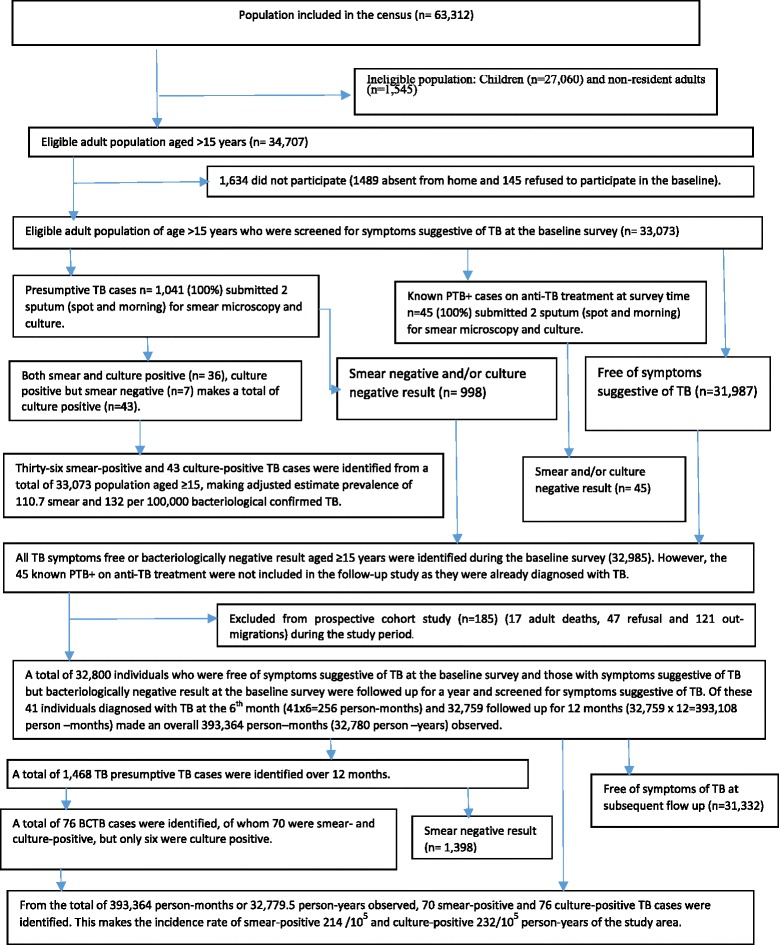
Study flow chart showing the study participants, screening procedure, sputum smear and culture results, Hetosa District, Arsi Zone of Oromia Region, Central Ethiopia 2016

A total of 63,312 individuals were enumerated during the pre-survey census (Fig. 1). Of these, 34,707 were eligible and thus participated in the prevalence survey. Of participated, 33,073 (95.3%) were screened for SSPTB. The average number of eligible individuals who participated from each cluster was 674.9. The mean age of the screened individuals was 33.3 years (standard deviation; SD 16.2) and the median age was 30.3 years. The overall response rate was 95.3%, with 95.8% for females and 94.8% for males. The overall participation rate was over the 90% expected in the study design. However, a higher participation rate among rural clusters (97.6%) was seen compared to the urban ones (85.9%). Out of the total of 34,707 eligible individuals, 1,634 (4.7%) did not participate in the prevalence survey. Of the latter, 1,489 (91.1%) were not at home, while 145 (8.9%) were not willing to participate in the survey (Table 1 and Fig. 1).

**Table 1:** Participation of eligible individuals in the prevalence survey, Hetosa District of the Arsi Zone of Oromia Region, Central Ethiopia

Variable	No. of eligible individuals identified through census and expected to participate	No. of eligible individuals who participated in the survey and were screened	Proportion of screened participants from the total eligible population
Sex			
Male	17,834	16,907	94.8
Female	16,873	16,166	95.8
Total	34,707	33,073	95.2
Age			
15–24	12,115	11,691	96.5
25–34	8,331	7,889	94.7
35–44	5,959	5,697	95.6
≥45	8,302	7,796	93.9
Mean age	33.3 (SD 16)		
Total	34,707	33,073	95.2
Stratum			
Rural	27,838	27,170	97.6
Urban	6,869	5,903	85.9
Total	34,707	33,073	95.2
PTB+ identified through passive case findings			
Male		28	Male to Female ratio
Female		17	
Total		45	1: 0.61
Number of BCTC identified by the survey			
Male		19	Male to Female ratio
Female		24	
Total		43	0.79:1

### Screening and sputum submission

A total of 33,073 eligible individuals were screened for SSPTB in the prevalence survey. Of these, 27,173 (82%) were rural residents and 16,907 (51.1%) were males. Moreover, a total of 32,800 individuals were enrolled for the follow-up study (Table 1). Of these, 31,802 were free of SSPTB at the baseline survey, while 998 showed SSPTB but yielded a bacteriologically negative result. A total of 1,041 and 1,468 individuals at the baseline survey and during the follow-up study, respectively, were reported to have SSPTB. These provided two sputum samples for bacteriological examination (AFB microscopic examination and culture). Of the former group, 258 (24.8%) individuals reported previous history of TB treatment, but were not on anti-TB treatment at the time of the survey. However, 45 known PTB+ cases were diagnosed through passive TB case findings and had been on anti-TB treatment at the time of the study. These also provided two sputum samples for bacteriological examination. Fig. 2 summarizes the screening and subsequent bacteriological examination results.

### TB cases identified

A total of 1,041 individuals with SSPTB and 45 PTB+ cases known to be on anti-TB treatment at the baseline survey provided spot and morning sputum samples for bacteriological examination. Of the 1,041 presumptive TB cases, 43 were found to have bacteriologically confirmed TB (culture and/or smear positive) whereas none of the 45 individuals on anti-TB treatment showed a positive result (Fig. 1).

The mean (SD) age of the diagnosed TB cases was 30 (10.3) years and the median was 30.8 years. Of the 43 bacteriologically confirmed TB cases, 36 were both smear- and culture-positive while seven were smear-negative but culture-positive. From the 36 smear-positive TB cases, 12 (33.3%) had a family history of TB contacts (Table 2).

**Table 2:** Prevalence of smear-positive and bacteriologically-confirmed pulmonary TB among population aged ≥15 years, Hetosa District of Arsi Zone of Oromia Region, Central Ethiopia

Category	Number of participants	Smear-positive Pulmonary Tuberculosis (PTB+)			Bacteriologically Confirmed Pulmonary Tuberculosis (BCTB)			
		Number of PTB+	Crude Prevalence estimate of PTB+ (95% CI)/100,000	Adjusted* prevalence estimate of PTB+ (95% CI)/100,000	Number of BCTB	Crude Prevalence Estimate of BCTB (95% CI)/100,000	Adjusted* Prevalence estimated of BCTB (95% CI)/100,000	Adjusted Odds Ratio
sex								
Male	16,907	16	89 (48.3–141.0)	89 (42.2–147.1)	19	112 (62.0–163)	112.2 (55.8–170.2.)	1.00
Female	16,166	20	124 (69.5–177.9)	124 (63.4–184.0)	24	149 (89.1–215.0)	148.5 (81.9–210.4)	1.3 (0.71–2.43)
Age								
15–24	11,691	5	43 (5.3–80.3)	43 (4.1–81.3)	8	68 (26.3–92.2)	68 (19.1–99.4)	1.00
25–34	7,889	12	152 (66.1–238.1)	152 (60.0–244.2)	13	165 (75.2–254.0)	165 (68.0–261.2)	3.4 (1.38–8.61)
35–44	5,697	11	193 (79.1–307.1)	193 (73.0–313.2)	11	193 (79.1–307.2)	193 (72.0–314.4)	4.2 (1.71–10.20)
≥45	7,796	8	103 (31.5–237.7)	103 (25.4–233.8)	11	141 (57.8–224.4)	141 (50.6–231.6)	2.76 (1.14–6.72)
Residence								
Urban	5,903	5	85 (10.5–159)	85 (4.4–165.1)	9	153 (104.2–254.0)	153 (97.0–261.2)	1,23 (0.59–2.57)
Rural	27,170	31	114 (73.9–154.2)	114 (67.5–160.3)	34	125 (97.3–224.4)	125 (90.1–231.6)	1.00
History of TB contact								
NO	31,170	24	77 (42.0–107.7)	77 (36.0–113.8)	31	98 (65.2–133.8)	98 (58.0–141.0)	1.00
Yes	1,867	12	643 (280.2–1005.2)	894 (274.1–1011.3)	12	643 (280.2–1005.2)	643 (273.0–1012.4)	13.0 (6.55–15.33)
Total	33,037	36	109 (73.3–144.3)	109 (67.2–150.5)	43	130.2 (91.2–169)	130.2 (83–176.2)	

NB. *Adjusted prevalence estimate of PTB+ and BCTB analysed using robust standard errors to account for the sample survey design

Furthermore, 32,800 individuals were enrolled in the follow-up study. Of these, 32,759 were followed for 12 months, making 393,108 person-months of observation, and 41 were diagnosed with TB at the end of the sixth month making 246 person-months of observation. Overall, a total of 393,354 person-months or 32,779.5 person-years (py) were observed. Of the total of 393,354 person-months or 32,779.5 py observed, 76 bacteriologically confirmed TB cases were identified (41 at the end of the sixth month and 35 at the end of the 12th month). Of these, 70 were smear- and culture-positive while the remaining six were smear-negative but culture-positive (Fig. 1 and Table 3).

**Table 3:** Study population, smear-positive TB cases identified over 12 months and incidence rate per 100,000 persons per year, Hetosa District, Arsi Zone of Oromia Region, Central Ethiopia

Category	Person-year	Smear-positive Pulmonary Tuberculosis (PTB+) (*n* = 70)			Bacteriologically Confirmed Pulmonary Tuberculosis (BCTB) (*n* = 76)		
		Number of diagnosed PTB+ cases	Incidence rate per 100,000 person-year (95% CI)	Adjusted Incidence Rate Ratio (aIRR) (95% CI)	Number of BCTB	Incidence rate per 100,000 person-year (95% CI)	Adjusted Incidence Rate Ratio (aIRR) (95% CI)
sex							
Male	16,741	36	215 (144.8–285.3)	1.00	38	227 (156.7–299.2)	1.00
Female	16,038.5	34	212 (140.7–283.2)	1.22 (0.78–1.93)	38	237 (161.6–312.3)	1.26 (0.80–1.97)
Age							
15–24	11,631.5	16	138 (70.2–205)	1.00	19	163(64.1–236.8)	1.00
25–34	7,865	8	102 (31.2–172.2)	1.30 (0.64–2.67)	9	114 (39.7–189.2)	1.51(0.73–3.15)
35–44	5,514	20	363 (203.7–521.7)	2.40 (1.18–4.55)	21	381(218.0–543.7)	2.76 (1.38–5.52)
≥45	7,769	26	335 (206.0–463.3)	1.66 (1.44–4.91)	27	348 (216.4–478.6)	3.05 (1.63–5.71)
Residence							
Urban	5,843.5	13	222 (101.5–343.4)	1.00	16	274(139.6–408.0)	1.00
Rural	26,936	57	212 (156.7–266.5)	1.23 (0.71–2.14)	60	223(166.4–279.10	1.25 (0.72–2.16)
History of TB contact							
NO	31,138	50	161 (116.1–205.1)	1.00	56	180(132.7–217.6)	1.00
Yes	1,641.5	20	1218 (686.4–1752.4)	4.11 (2.18–7.77)	20	1218(686.4–1752.4)	5.11 (2.63–9.96)
Total	32,779.5	70	214 (163.5–263.5)		76	232(179.7–283.9)	

### Prevalence

The adjusted prevalence estimate of PTB+ individuals among adults aged ≥15 years was 109 (95% CI: 67.2–150.5) whereas that of BCTB was 132 (95% CI: 83.0–176.2) per 100,000 population. Even so, the adjusted prevalence of BCTB was higher among females (148.5) (95% CI: 81.1–210.4) than males (112.2) (95% CI: 55.8–170.2) but had no statistical difference (Table 2).

In the multivariate logistic regression model, age and family history of TB contacts were independently associated with high rates of PTB+ and BCTB cases. Compared to individuals in the age group from 15–24 years, those in the age group from 25–34 years were 3.4 times more likely to have TB [AOR: 3.4 (95% CI: 1.4–8.6)]. Those in the age group of 35–44 years were 4.2 times more likely [AOR: 4.2 (95% CI: 1.7–10.2)] while those ≥45 years were 2.7 times more likely to have the disease [AOR: 2.7 (95% CI: 1.1–6.7)]. The prevalence of TB therefore increased with age up to 44 years but declined from 45 years onward. Presumptive TB for those who had a family history of contact with TB patients was 13 times more likely than those without such a history [OR = 13.0, (6.5–25.3)].

The active and passive TB case findings of the study area were compared using the number of cases identified by each method. Forty-three BCTB cases were identified through current active TB case findings while 45 PTB+ cases on anti-TB treatment at the time of the survey were detected through passive case findings. Of the 45 PTB+ cases, 28 were males while 17 were females making a ratio of 1:0.61 (28/17). The male to female ratio for those identified through active case findings was 0.79:1 (19/24). The ratio of passive to active case findings was 1:0.96 (45/43) (Table 1).

### Incidence of smear-positive TB

From the total of 393,354 person-months (32,779.5 py) observed, 76 BCTB cases were identified. Of these, 70 were both smear- and culture-positive, while six were smear-negative but culture-positive. The incidence rate of PTB+ among adult individuals aged ≥15 years was 214 (95% CI: 163.5–263.5) whereas that of BCTB was 232 (95% CI: 179.7–283.9)/100,000 py in the study area (Table 3). There were 17 adult deaths, 47 refusals and 121 out-migrations during the study period and these were excluded from the analysis. Moreover, because the TB status of 1,634 individuals who did not participate in the baseline survey was not known, they were also excluded from the follow-up study (Fig. 1).

The incidence of PTB+ cases among males was 215 (95% CI: 144.8–285.3) whereas it was 212 (95% CI: 140.7–283.2) among females per 100,000 py. Likewise, the incidence of PTB+ per 100,000 py among urban residents was 222 (95% CI: 101.5–343.4) while it was 212 (95% CI: 156.7–266.5) among rural people. However, the difference in the incidence rates among males and females (adjusted incidence rate ratio) [aIRR, 1.22 (95% CI: 0.78–1.93], and among urban and rural dwellers [aIRR, 1.23 (95% CI: 0.71–2.14)] was not statistically significant (Table 3).

In the multivariate Poisson regression model, the age and history of TB contact were independently associated with a high risk of TB. Presumptive TB cases in the age group from 35–44 years were 2.4 times [aIRR, 2.40 (95% CI: 1.18–4.55)] more likely to have TB compared to those in the younger age group from 15–24 years. Compared to the same age group, those aged ≥45 were 2.7 times [aIRR 2.66 (95% CI: 1.44–4.91)] more likely to develop the disease. Presumptive TB cases who were either free of SSPTB or showed negative bacteriological examination results at the baseline survey but had history of contact with TB patients in the family were four times more likely to have TB than those with no history of such contact [aIRR, 4.11 (95% CI: 2.18–7.77)] (Table 3).

## Discussion

This population-based study identified a high prevalence and incidence of PTB+ and BCTB among individuals aged ≥15 in Hetosa District of Arsi Zone. For every TB case of PTB+ on treatment, there was an almost equal number (0.96) of undetected bacteriologically confirmed infectious TB cases in the community. The overall crude prevalence point estimate of PTB+ and BCTB cases was very similar to the inverse probability weighting prevalence point estimate using robust standard errors to account for the cluster survey sample design effect. Even so, there was a difference in precision with a wide confidence interval for the adjusted prevalence point estimate. The adjusted prevalence estimate of PTB+ and BCTB cases in the study area was 109 (95% CI: 67.2–150.5) and 130.2 (95% CI: 83.0–176.2)/100,000 population, respectively. In the follow-up study, the incidence of PTB+ and BCTB was 214 (95% CI: 163.3–263.5) and 232 (95% CI: 179.7–283.9)/100,000 py.

The 130.2/100,000 adjusted prevalence estimate of BCTB cases identified in this study is higher than the 34/100,000 reported from China and the 76/100,000 from Southwest Ethiopia [12]. Nonetheless, it is lower than the 169/100,000 report from Northern Ethiopia [27] and the 278/100,000 from the Lao PDR [28].

Moreover, the 109/100,000 adjusted prevalence of PTB+ cases in this study is similar to the 108/100,000 report of the national estimate [29]. Conversely, it is higher than previous reports that ranged from 30 to 80/100,000 population in different parts of the country [12, 14, 30], the 90/100,000 from Eritrea [31] and the 95/100,000 from Bangladesh [32]. Still, it is lower than the145/100,000 population reported from Vietnam [33], and the 169/100,000 population reported from Northern Ethiopia [27] and India [34].

The difference in the prevalence of BCTB and PTB+ TB cases across different geographic settings might be due to differences in the populations studied, the timing of the study or differences in the sampling, data collection and screening methods used across the different studies. For example, in some studies [12, 14, 30] the heads of households were interviewed to give testimony about the TB symptomatic cases of all family members in the household. However, the heads of households may not have sufficient information about all individual members while others interviewed all members of a household to screen presumptive TB cases [13, 29].

Some community-based TB prevalence studies used clinical diagnoses and chest x-rays before taking sputum for screening [29, 35, 36] while others, including the current study, used TB symptom-screening questionnaires [12–14, 36] to identify the cases. Nonetheless, the chances of detecting TB cases among non-symptomatic individuals increased by 20–50% when a combination of a TB symptom-screening questionnaire and a clinical diagnosis with a chest x-ray was employed, compared to using a TB symptom-screening questionnaire alone without a chest x-ray [7, 28, 29, 35, 37]. Hence, the adjusted estimate of BCTB prevalence in this study might be underestimated due to the fact that the chest x-ray screening method was not used to identify non-symptomatic TB cases.

The age group in the survey also varied across different studies. Some covered all age groups ≥15 years [29, 30, 32, 38] while others included those aged >14 years [14] and still others those aged 14 years [12, 13]. Consequently, a comparison of TB prevalence rates among studies within a country or elsewhere should be taken with caution.

The high prevalence of BCTB cases among younger age groups in this study is in agreement with a previous report [29], whereas the high prevalence of TB among the younger population may suggest ongoing TB transmission in the community. The prevalence of TB increased with age among the younger age groups up to the age of 45 years. However, a high TB incidence rate was observed among the older age group. This might be due to the high number of infectious TB cases identified at baseline, which could reduce the ongoing TB transmission among the general population, while the high TB incidence among the elderly is probably indicative of a latent TB reactivation among the older age group [13, 14, 29, 39]. Nevertheless, further study is required to fully understand why the observed high TB prevalence among younger individuals also corresponded to a high incidence in the older age group.

In this study, 43 BCTB cases were identified through active TB case findings at the baseline survey while 45 PTB+ cases were identified through existing passive TB case findings. Thus, the ratio of PTB+ cases being treated at the time of the survey to newly detected BCTB cases was 1:0.96 suggesting that for every PTB+ case receiving treatment during the survey, there was an almost equal number (0.96) of cases of BCTB existing in the community. This indicated that there was a very high proportion of undiagnosed infectious TB cases present in the community. In Southern Ethiopia, there were two cases [14] while in South Africa there were 4.5 cases [40] of passive detection for every TB case identified through active case findings. In Northern Ethiopia, the ratios of passive to active TB case findings were 2.5:1 [30] and 2:1 [13]. This implies that there is a high number of undiagnosed infectious TB cases in the present study area compared to reports by previous studies. The high proportion of undetected infectious TB cases in the community might be due to the sub-optimal DOTS performance in identifying 70% of infectious TB cases and attaining the global target of 85% cure rate in Ethiopia [41–43].

Moreover, the difference in the number of undetected infectious TB cases across different geographic settings might be attributed to variation in DOTS performance, DOTS service coverage and the quality of DOTS services across various study areas. It could also be attributed to the difference in DOTS service uptake that might result from differences in public awareness about TB. Consequently, the decentralization and strengthening of the community in TB care could help to pick up undetected infectious TB cases in Arsi Zone.

The male-to-female ratio among PTB+ cases identified through existing passive TB case findings was 1:0.61 (28/17) whereas the ratio among BCTB cases identified by the current active TB case finding was 0.79:1 (19/24). This may indicate a lower rate of passive case findings among females compared to males. The lower passive and higher active TB case findings among women in this study is in agreement with reports from Southern [14] and Northern Ethiopia [13, 27], Bangladesh [44] and India [34] where more women were identified through active TB case findings. The lower passive TB case findings among females might be due to poor access to health services, and as shown in a study conducted in South Africa, women are less likely to be asked for a sputum sample when they appear at health facilities [45]. Moreover, their economic dependence and low health-care seeking behaviour possibly hindered women from visiting health institutions to obtain TB care services. Barriers to accessing health services among TB patients and a failure to detect women with TB through the routine TB control programme warrant further inquiry.

As expected, history of TB contact increased the risk of having active TB. A recent systematic review and previous reports have shown that history of TB contact was associated with a high risk of TB [30, 46–48]. Findings by the current study are in line with those of a systematic review and large epidemiological surveys that have established the association between history of TB contact and higher risk of TB [47, 49, 50]. Therefore, contact-tracing efforts should target households with members who are PTB+ so as to capture the undetected infectious TB cases in the community.

In this study, the high prevalence of BCTB cases in urban areas confirms previous reports of high TB prevalence in urban settings [16, 51, 52]. In contrast, the national prevalence TB survey reported higher TB prevalence among dwellers in the rural areas [29]. This is due to the inclusion in the national prevalence survey of pastoralists in the rural population where the highest prevalence ratio of 170/100,000 was observed [21] as opposed to the current study. The pastoralist population may have poor access to TB care, as well as low awareness and health-seeking behaviour which might have resulted in them having a high burden of undiagnosed TB cases and eventually elevating the prevalence of TB among the rural population in the national prevalence survey. The higher prevalence of BCTB cases among urban settings compared to rural areas in the current study may be due to the overcrowded living conditions and dichotomy of higher HIV prevalence in the urban areas of the country.

The 214/100,000 py incidence rate of PTB+ cases in this study is similar to the 212/100,000 py reported from South Africa [53]. However, it is higher than the 197/100,000 py from Guinea-Bissau [54] and the 207/100,000 py from Southern Ethiopia [15]. Nonetheless, it is lower than the 311/100,000 py reported from Northern Ethiopia [11]. The high incidence of TB in the present study might be an indication of the ongoing transmission of the disease that might result from a sub-optimal DOTS performance in the interruption of TB transmission. For instance, according to previous reports, there was a low rate of PTB + case detection rate (37.7%) [41] and a low cure rate (66.9%) [42] and a high prevalence of drug resistance TB [55] in the study area. Hence, the low PTB+ case detection rate, cure rates and high drug resistance TB reported from the study area, combined with the high prevalence and incidence rates identified by the current study, may confirm the sub-optimal performance of DOTS in curbing the active transmission of TB. Therefore, the involvement of health extension workers in educating the community on TB as well as accelerating referral of presumptive TB cases may improve the possibility of capturing undiagnosed infectious TB cases in the community.

Information on the prevalence and incidence of TB is a valuable epidemiological indicator to help assess the impact of national and international TB control efforts. Nevertheless, community-based data on BCTB prevalence and incidence are lacking in developing countries including Ethiopia. As a result, the findings of this study with regard to the prevalence and incidence rates of BCTB cases are among the very few population-based studies in resource-poor settings.

In this study, efforts were made to maintain the quality of the study, and rigorous training was given for data collectors and laboratory technicians. The study population was monitored to identify deaths and migrations during the prospective follow-up study to provide an accurate time contribution in the denominator to compute the incidence rate. Moreover, we have used very sensitive standardized and pre-tested questionnaires to screen presumptive TB cases experienced and qualified laboratory technicians to carry out smear microscopy and sputum culture. Following that, a senior laboratory technologist who was blinded to the results of the first test results re-examined all the smear-positive, and 10% of the smear-negative slides, to validate the quality of laboratory results. Additionally, the estimated design effect we have used in this study was 2 whereas the actual calculated design effect from the current study data was 1.3 there by indicating that the sample size of our study was adequate and is representative of the study population of the district. Likewise, in order to obtain the adjusted precision of PTB+ and BCTB prevalence of the study, the design effect for the cluster sample survey was taken into account during the analysis.

On the other hand, although our study was among the very few attempts to detect a community-based TB incidence and may contribute valuable information to the TB control programme in Ethiopia, it has some limitations. First, SSPTB was used as screening mechanism. The fact that chest x-ray was not used in our study might underestimate the prevalence and incidence of TB in the area. The missing diagnosed TB cases at the baseline survey but which were later included in the prospective study might have resulted in an over-estimation of the TB incidence rate. Second, we excluded 20 contaminated sputum cultures from the analysis at the baseline survey and this may also have led to an underestimation of the prevalence of sputum culture TB. Third, we carried out a survey three times, first at the beginning of the study to determine the prevalence of TB, followed by the second at the end of the sixth month and the third at the end of the 12th month to estimate incidence of TB. However, the six-month time interval between surveys may have given sufficient time for spontaneous self-cure of active TB cases, which might have led to underestimation of the true incidence of TB cases in the study area. Fourth, we excluded 1,634 individuals who did not participate in the baseline survey from the subsequent follow-up study. Moreover, 47 individuals who refused to participate in the follow-up study plus 18 deaths and 119 out-migrants were excluded from the analysis of the incidence rate due to the fact that the exact time of their contribution to the denominator was not known. Nonetheless, the overall proportion of participants excluded was only 5.5% and their baseline socio-demographic characteristics were similar to those included in the analysis. Therefore, their exclusion may not affect the overall findings of the study. Fifth, although HIV is a known risk factor for TB, we did not screen presumptive TB cases for HIV to measure the impact of HIV in fueling TB in the study area. Sixth, some relevant variables that might have affected the outcomes of interest were not included in the study. Hence, stratifying and analyzing only those included variables is less likely to fully control for other possible confounding variables and may introduce bias.

## Conclusions and recommendations

The prevalence and incidence of smear-positive and bacteriologically confirmed TB cases were high in the study area. For every case of smear-positive TB receiving treatment, there was an almost equal number (0.96) of undetected infectious bacteriologically confirmed TB cases in the community. The high proportion of undetected infectious TB cases in the community could have resulted from the sub-optimal DOTS performance in detecting 70% of infectious TB cases and attaining a cure rate of 85% in the study area. For this reason, there is a need to design an alternative strategy to improve TB case findings. A family history of contact has substantaially increased the risk of developing the disease, so there is a need to improve the identification of TB cases and intensify contact tracing among household members of PTB+ cases through the involvement of community-based health extension workers.
